# Traction Sural Neuropathy Following Nonoperative Management of Acute Achilles Tendon Rupture: A Case Report

**DOI:** 10.7759/cureus.74420

**Published:** 2024-11-25

**Authors:** Katherine J Kim, Isabella V Cuturrufo, Kenneth Vitale

**Affiliations:** 1 Department of Orthopedic Surgery, Division of Sports Medicine, University of California San Diego School of Medicine, La Jolla, USA

**Keywords:** achilles tendon rehabilitation, achilles tendon rupture, mechanical traction injury, nonoperative management, sports medicine, sural neuropathy, tendon healing

## Abstract

Achilles tendon ruptures are prevalent among physically active adults and can lead to sural nerve injuries (SNIs) due to the anatomical proximity of the sural nerve to the Achilles tendon. While SNIs are well-recognized in surgical contexts, their occurrence following nonoperative treatments, which are often preferred for their lower risk of surgical complications, remains less documented and poorly understood. This report describes a case of a 30-year-old active male who developed chronic traction sural neuropathy after opting for nonoperative treatment of an acute complete Achilles tendon rupture. Despite adhering to a rehabilitation protocol, he experienced persistent symptoms of sural nerve damage, which were confirmed as chronic sensory neuropathy through nerve conduction studies. Here, we discuss the proposed pathophysiology and review the literature on SNIs in Achilles injuries. This case highlights the importance for clinicians to accurately diagnose and remain aware of the potential for SNIs in the nonoperative management of Achilles tendon ruptures.

## Introduction

Achilles tendon ruptures are among the most prevalent tendon injuries, especially in active adults. The sural nerve, which provides cutaneous sensation to the lateral aspect of the lower leg and foot, is at risk of injury in an Achilles tendon rupture due to its anatomic proximity to the tendon [1,2]. While sural nerve injury (SNI) is a recognized complication of surgical repair, its incidence and implications in nonoperative treatments are less documented and understood [1,3].

Nonoperative strategies for treating an Achilles tendon rupture are often chosen for lower-risk cases or patients who are not suitable candidates for surgery. These approaches, which rely heavily on the tendon’s natural healing ability, avoid potential surgical complications, such as infection or direct nerve damage [4]. However, even in the absence of direct surgical trauma, the sural nerve can potentially be affected indirectly due to proposed traction injury or changes in local anatomy and inflammation caused by an acute ankle injury itself. In the case of an Achilles tendon rupture, sural neuropathies may result from mechanical stretch or compression beneath the fascial planes adjacent to the Achilles tendon during the acute phase of injury [5]. These neuropathies can persist chronically, complicating or delaying full recovery and impacting patient outcomes negatively [6].

Given the significant functional impairment that can result from SNI, it is important for clinicians to understand its pathophysiology in both surgical and nonsurgical treatments of Achilles tendon injuries. This case report describes an unusual presentation of chronic sural neuropathy following the nonoperative management of a complete Achilles tendon rupture, highlighting the need for heightened awareness of SNI in conservative treatments.

## Case presentation

A 30-year-old male salesman and rugby coach presented to urgent care with sudden pain and inability to bear weight on his right leg following a soccer injury. While running, he reported a sensation akin to being kicked in the back of his right leg, although no actual contact occurred. His past medical history included two right knee arthroscopies for a medial meniscus tear but no other significant medical or family history relevant to his current condition. Initial evaluation at urgent care found swelling in the distal calf and a palpable defect in the Achilles tendon area. The patient was prescribed oral diclofenac for pain, provided with a CAM walking boot, and advised to use crutches for non-weight-bearing mobility until he could consult with a specialist.

**Figure 1:**
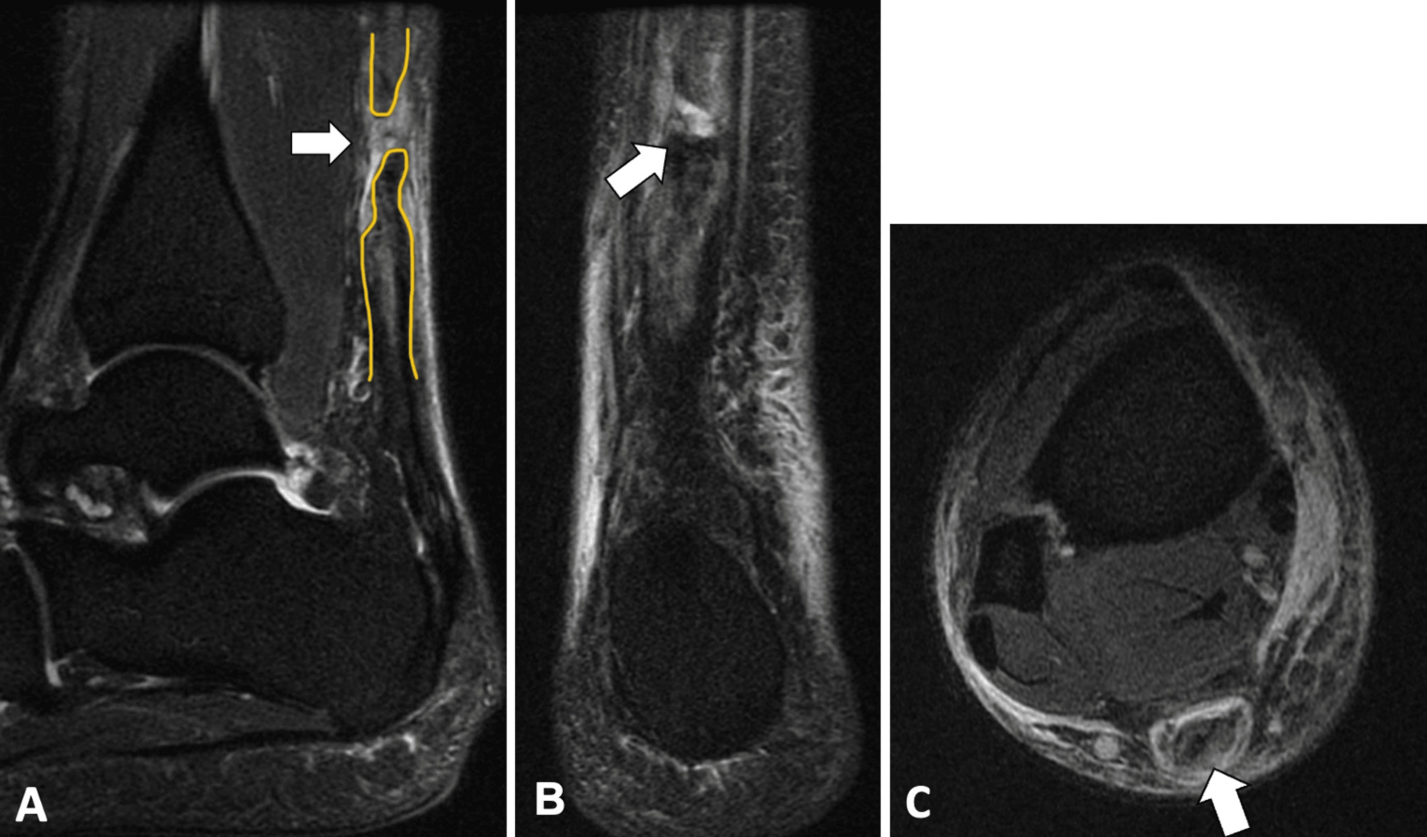
MRI without contrast of the right foot/ankle T2-weighted fat-suppressed MRI showing a complete tear of the Achilles tendon. A: sagittal section; B: coronal section; C: axial section

Several days after the initial injury, the patient visited a podiatrist who confirmed a complete right-sided Achilles tendon rupture via multiplanar and multisequence magnetic resonance imaging (MRI) without contrast (Figure 1). MRI of the patient’s right ankle showed a complete tear of the Achilles tendon, approximately 84 mm above its calcaneal insertion, with less than 1 cm of retraction of the torn tendon fibers, and closely aligned tendon edges. Clinical examination revealed weakness in plantarflexion, positive Thompson and Malte’s tests, decreased sensation along the posterolateral lower leg, and tenderness and swelling several centimeters proximal to the calcaneal insertion in the posterior right lower leg. At this visit, the patient’s CAM boot was adjusted with added heel lifts and wedges for better support and comfort.

The patient subsequently consulted with an orthopedic surgeon to discuss both surgical and nonsurgical treatment options for his complete Achilles tendon rupture. Given the recent occurrence of the injury and MRI findings showing tendon edges in close proximity, it was explained to the patient that his injury was likely to heal well with a nonoperative functional rehabilitation protocol. Additionally, it was noted that surgery might be technically challenging due to the proximal location of the rupture, which left limited tendon available for suture fixation.

Following this discussion, the patient was initially undecided on his choice of treatment. However, after reviewing the comparative risks and benefits and reflecting on his personal goals and concerns about surgical complications, he decided to proceed with nonoperative management. The patient was then placed on an accelerated functional rehabilitation protocol, which included progressive weight-bearing and mobility exercises (Table 1).

**Table 1:** Achilles tendon rupture accelerated functional rehabilitation protocol

Time Period	Rehabilitation Protocol
Weeks 0-2	Splint in plantarflexion or CAM boot with 2 cm heel lift; non-weight-bearing with crutches
Weeks 2-6	Tall CAM boot with 2 cm heel lift; protected weight-bearing (WB) with crutches as required; active plantar and dorsiflexion to neutral, inversion/eversion below neutral; modalities to control swelling; knee/hip exercises with no ankle involvement as appropriate; non-weight-bearing fitness/cardio work; hydrotherapy (within motion and weight-bearing limitations)
Weeks 6-8	Aircast boot or CAM boot; discontinue 1 heel lift each week; transition to WB as tolerated; dorsiflexion stretching slowly; graduated resistance exercises (open and closed kinetic chain as well as functional activities); proprioceptive and gait retraining; modalities including ice, heat, and ultrasound as indicated; fitness/cardio to include weight-bearing as tolerated; hydrotherapy
Weeks 8-12	Wean off boot; return to crutches/cane as necessary and gradually wean off; continue to progress a range of motion (ROM), strength, proprioception
Weeks >12	Continue to progress ROM, strength, and proprioception; retrain strength, power, and endurance; increase dynamic WB exercise, including plyometric training; sport-specific retaining

The patient experienced a delayed start in his rehabilitation due to initial conservative deviations from the prescribed protocol by his physical therapist. Despite this slow start, he demonstrated significant improvement in his follow-up visits throughout the year. At six weeks, he advanced to weight-bearing as tolerated, and by three months, he transitioned from his CAM boot to regular footwear. At approximately 33 weeks post-injury, he was capable of performing box jumps, cycling, and plyometric exercises, and reported increased strength in his tendon. However, he also reported persistent numbness in the lateral hindfoot, which affected his ability to receive feedback during activities and exercise. This prompted further evaluation through electrodiagnostic testing, including a nerve conduction study (NCS) and needle electromyography (EMG).

**Figure 2:**
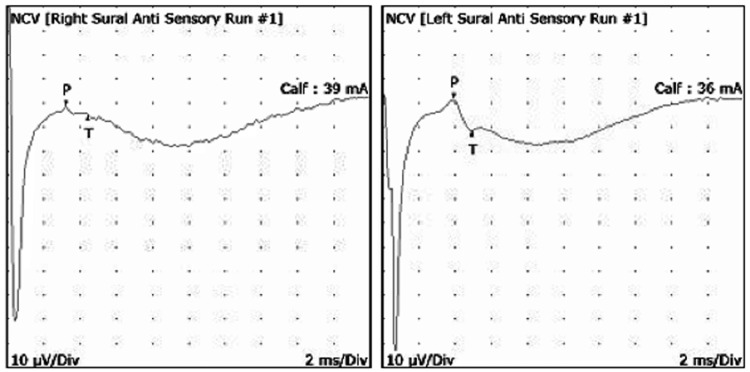
Sample NCS waveform recording showing a right and left comparison of the sural nerve responses The right sural nerve amplitude potential was reduced (3.2 µV) compared to a normal left amplitude (13.0 µV).

NCS indicated an isolated chronic axonal right sural sensory neuropathy, marked by a significantly reduced signal amplitude in the right sural sensory nerve (3-5 µV) compared to the normal left side (13-13.4 µV) with a side-to-side amplitude difference of 72.8% (Tables 2-4, Figure 2). Incidentally, the right peroneal motor nerve recording at the extensor digitorum brevis (EDB) also displayed a low onset-to-peak amplitude of 0.6 mV, which is 53.9% less than the expected baseline, most likely due to disuse atrophy from prolonged boot immobilization (Table 4). There was no indication of peripheral polyneuropathy, L5 or S1 radiculopathy, or peroneal or tibial entrapment neuropathy. Needle EMG showed no active denervation but again noted signs of atrophy in the EDB (Table 5).

**Table 2:** Sensory testing nerve conduction study results of the lower extremity P-T, peak-to-trough; Amp, amplitude

		Right		Left		Left-Right Difference (%)		Normal	
Nerve	Stimulation Site	Peak (ms)	P-T Amp (µV)	Peak (ms)	P-T Amp (µV)	Peak (ms)	P-T Amp (µV)	Peak (ms)	P-T Amp (µV)
Superficial peroneal (run #1)	Anterior lateral malleolus	4.0	11.6					<4.4	>5.0
Superficial peroneal (run #2)	Anterior lateral malleolus	4.3	6.8					<4.4	>5.0
Sural (run #1)	Lateral malleolus	3.2	3.2	3.9	13.0	19.7%	121.0%	<4.5	>4.0
Sural (run #2)	Lateral malleolus	2.9	5.0	4.3	13.4	38.9%	91.3%	<4.5	>4.0
Sural (run #3)	Lateral malleolus	2.9	3.9					<4.5	>4.0
Sural (run #4)	Lateral malleolus	3.9	3.5					<4.5	>4.0

**Table 3:** Left-right comparison of sural nerve responses Amp, amplitude; L, left; R, right; Lat, latency

Nerve	Stimulation Site	L Lat (ms)	R Lat (ms)	L-R Lat (ms)	L Amp (µV)	R Amp (µV)	L-R Amp (%)	Site 1	Site 2
Sural	Lateral malleolus	4.3	3.9	0.4	13.4	3.65	72.8%	Calf	Lateral malleolus

**Table 4:** Motor testing nerve conduction study results of the lower extremity Amp, amplitude; O-P, onset-to-peak

Nerve	Stimulation Site	Onset (ms)	Normal Onset (ms)	O-P Amp (mV)	Normal O-P Amp (mV)	Velocity (m/s)	Normal Velocity (m/s)
Right peroneal motor	Ankle	0.9	<6.5	0.6	>1.3		
Right tibial motor (#1)	Ankle	3.7	<6.1	12.6	>4.4		
Right tibial motor (#2)	Knee	13.7	<6.1	12.6	>4.4	46	>39

**Table 5:** Results of needle electromyography of the right lower extremity Ins Act, insertional activity; Fibs, fibrillation potential; Psw, positive sharp wave; Amp, amplitude; Dur, duration; Poly, polyphasia; Recrt, recruitment; Int Pat, interference pattern; Nml, normal

Muscle	Nerve	Root	Ins Act	Fibs	Psw	Amp	Dur	Poly	Recrt	Int Pat
Anterior Tibialis	Deep Peroneal	L4-5	Nml	Nml	Nml	Nml	Nml	0	Nml	Nml
Peroneus Long	Superficial Peroneal	L5-S1	Nml	Nml	Nml	Nml	Nml	0	Nml	Nml
Medial Gastrocnemius	Tibial	S1-2	Nml	Nml	Nml	Nml	Nml	0	Nml	Nml
Biceps Femoris	Sciatic	L5-S1	Nml	Nml	Nml	Nml	Nml	0	Nml	Nml
Vastus Medialis	Femoral	L2-4	Nml	Nml	Nml	Nml	Nml	0	Nml	Nml
Abductor Digiti Quinti	Lateral Plantar	S1-2	Nml	Nml	Nml	Nml	Nml	0	Nml	Nml
Abductor Hallucis	Medial Plantar	S1-2	Nml	Nml	Nml	Nml	Nml	0	Nml	Nml
Extensor Digitorum Brevis	Deep Peroneal	L5-S1	Low	Nml	Nml	Nml	Nml	0	Nml	50%
Extensor Digitorum Longus	Deep Peroneal	L5-S1	Nml	Nml	Nml	Nml	Nml	0	Nml	Nml
Soleus	Tibial	L5-S2	Nml	Nml	Nml	Nml	Nml	0	Nml	Nml

**Figure 3:**
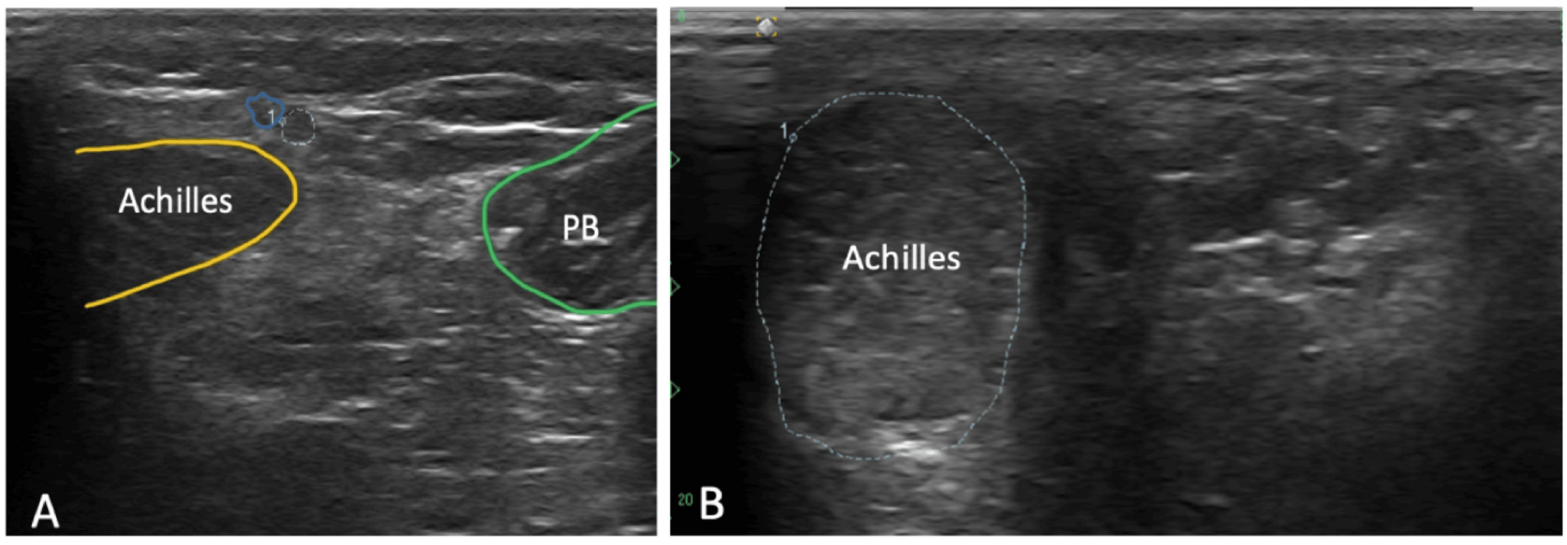
Ultrasound findings of the right ankle A: Short-axis view of Achilles tendon (yellow), small saphenous vein (solid blue), sural nerve (dotted blue), and peroneus brevis (green); B: Transverse view showing a thickened Achilles tendon (dotted blue) PB, peroneus brevis

Ultrasound of the right ankle showed a small, visible sural sensory nerve in cross-sectional views proximally. This became difficult to visualize at the level of the tear, clinically corresponding to the patient's reported site of numbness (Figure 3). Comparative imaging of the left side revealed a normal caliber left sural sensory nerve that was traceable distally into the ankle. Notably, there was no evidence of a gross Achilles tear; however, scar tissue, fibrosis, and a thicker diameter of the Achilles tendon were observed on the right.

The orthopedic surgeon discussed the implications and initially suggested a referral to a peripheral nerve surgery specialist if symptoms were to persist. However, since the patient was making progress in his activities, it was decided to continue with observation and ongoing physical therapy. At his last follow-up, approximately 46 weeks post-injury, the patient had significant improvement in his Achilles tendon strength and functionality, managing to perform both double and single-leg heel raises and hopping on his affected leg. He reported improved overall strength and felt nearly restored to his pre-injury level of physical activity, although he remained cautious about returning to sports. While he still experienced mild sensory deficits in the sural nerve distribution, particularly over the lateral aspect of the heel, his nerve symptoms were gradually improving. Clinical examination confirmed intact gross sensation to light touch. The decision was made to continue observation of his neuropathy, with periodic evaluations to monitor progress.

## Discussion

Achilles tendon rupture is a significant injury commonly seen in individuals actively involved in sports. This injury, which can be a partial or complete tear, typically presents with sudden, sharp pain, often described as feeling like a kick to the back of the leg, mirroring our patient’s experience [7,8]. The choice between surgical or nonsurgical treatment depends on several factors, such as the patient's age, activity level, and the severity of the tear.

In this case report, we presented a 30-year-old male who sustained a complete Achilles tendon rupture and opted for nonoperative management. Despite avoiding the risks associated with surgery, he developed chronic sural neuropathy, a complication more commonly linked to surgical interventions due to direct nerve exposure and the potential for damage during the procedure. This unusual presentation illustrates that sural nerve complications can also arise from nonoperative management, likely caused by nerve compression or mechanical traction associated with the injured tendon [4].

The existing literature largely debates the pros and cons of surgical versus nonsurgical treatments for an Achilles tendon rupture. Surgical repair is often favored for its lower re-rupture rates but comes with higher risks of complications, such as infections and nerve injuries [9]. Conversely, nonoperative management has been shown to yield comparable functional outcomes with fewer initial complications [10-12]. One randomized controlled trial found that, in patients with an acute Achilles tendon rupture, surgery (open repair or minimally invasive) did not result in better outcomes than nonoperative treatment at 12 months based on the Achilles Tendon Total Rupture Score [10]. Nonsurgical management may predispose patients to mechanical disruptions or stretching of the sural nerve, influenced by swelling, scarring, or altered limb mechanics during the healing process, which are factors that are less controllable than in surgical treatments [13].

Studies have noted the extremely rare occurrence of SNI in patients managed nonoperatively, with systematic reviews and meta-analyses reporting an incidence rate of less than 1% [10,11,14]. Historically, prior to 1980, only six cases of sural nerve entrapment or compression were reported in PubMed, associated with conditions such as ganglia, Baker's cyst, or posttraumatic scarring, with none attributed to stretch injuries [15-17]. In 2000, a case series reported 13 athletes with sural nerve entrapment, suspected to be due to increased sural muscle mass from intensive physical training, but not from Achilles tendon injury [18]. To our knowledge, there are a total of two cases of sural nerve injuries that have been reported in patients with delayed presentation of a ruptured Achilles tendon [19].

It is possible that SNIs are underreported due to their symptoms, which can resemble conditions such as radiculopathy, or patients may confound sural nerve neuropathic symptoms with the Achilles injury itself. This may lead to potential misdiagnosis or oversight. This case highlights the potential for an associated SNI in the nonoperative management of an Achilles tendon rupture. Clinicians should diligently monitor neurological symptoms, such as numbness and weakness, in all patients with Achilles tendon injuries, regardless of the chosen treatment method. Regular neurological assessments can help identify nerve injuries early, allowing for appropriate management and enhanced patient outcomes.

## Conclusions

This case report illustrates the potential for chronic sural neuropathy following nonoperative management of Achilles tendon ruptures. It challenges the assumption that conservative treatments are devoid of significant complications and highlights the importance of integrating regular neurological evaluations into rehabilitation protocols to mitigate the risks of SNIs. The goal would be to identify and address such complications promptly. By doing so, clinicians can better personalize management plans that address both the structural healing of the tendon and the patient's overall sensory and functional recovery, which may potentially lead to better outcomes and enhanced patient satisfaction.
